# A Screen for Recessive Speciation Genes Expressed in the Gametes of F1 Hybrid Yeast

**DOI:** 10.1371/journal.pgen.0030021

**Published:** 2007-02-16

**Authors:** Duncan Greig

**Affiliations:** Department of Biology, University College London, London, United Kingdom; Princeton University, United States of America

## Abstract

Diploid hybrids of Saccharomyces cerevisiae and its closest relative, *Saccharomyces paradoxus,* are viable, but the sexual gametes they produce are not. One of several possible causes of this gamete inviability is incompatibility between genes from different species—such incompatible genes are usually called “speciation genes.” In diploid F1 hybrids, which contain a complete haploid genome from each species, the presence of compatible alleles can mask the effects of (recessive) incompatible speciation genes. But in the haploid gametes produced by F1 hybrids, recessive speciation genes may be exposed, killing the gametes and thus preventing F1 hybrids from reproducing sexually. Here I present the results of an experiment to detect incompatibilities that kill hybrid gametes. I transferred nine of the 16 S. paradoxus chromosomes individually into S. cerevisiae gametes and tested the ability of each to replace its S. cerevisiae homeolog. All nine chromosomes were compatible, producing nine viable haploid strains, each with 15 S. cerevisiae chromosomes and one S. paradoxus chromosome. Thus, none of these chromosomes contain speciation genes that were capable of killing the hybrid gametes that received them. This is a surprising result that suggests that such speciation genes do not play a major role in yeast speciation.

## Author Summary

A species is usually defined as such because it cannot exchange its genes with other species. Closely related species may attempt to breed but be unsuccessful. A common example of this occurs when a donkey mates with a horse. The offspring of this mating is a hybrid called a mule. Mules are sterile and cannot reproduce, so donkeys and horses are maintained as distinct species—they cannot exchange genes. Understanding what makes hybrids sterile could tell us how new species originate. Instead of mules, this study examines yeast hybrids that are sterile because the sex cells (the yeast equivalent of sperms or eggs) they produce are dead. One possible reason for this is that the genes from the different species fail to work together in the sex cells, killing them. To test this idea, I replaced individual chromosomes in one species' sex cells with chromosomes from another species. Surprisingly, this did not kill the gametes, showing that the genes from one species can work fine with the genes of another. Not all the genes could be tested in this way, but nevertheless it seems likely that the death of sex cells produced by yeast hybrids is caused by something other than failure of the genes from different species to work together.

## Introduction

Hybrid sterility is important because it prevents genes from being exchanged between species that interbreed and hence maintains species integrity. It is widely accepted that hybrid sterility can be caused by incompatibility between genes from different species. Dobzhansky [1] and Muller [2] described how geographically separated populations would fix beneficial alleles at different loci that could be incompatible if the populations were reunited and formed hybrids. Genetic incompatibilities that sterilize or kill hybrids are often called “speciation genes” [3], although such incompatibilities would eventually evolve anyway even if speciation was caused by something else. Genetic analyses are therefore best performed on recently formed species, in which incompatible genes are more likely to be a cause, rather than a consequence, of speciation. There are many such analyses in *Drosophila* hybrids, which typically have large numbers of incompatibilities. For example, male hybrids of Drosophila mauritania and Drosophila simulans are sterile and probably have approximately 100 genetic incompatibilities [4,5], including the celebrated gene *Odysseus* [6]. Speciation genes have also been identified in other taxa. For example, simple genetic incompatibility between two loci causes hybrids of the platyfish and swordfish to develop melanoma [7] and monkey flower hybrids to die as embryos [8].

The baker's yeast *Saccharomyces cerevisiae* forms diploid F1 hybrids with the wild yeast *S. paradoxus.* Hybrids can reproduce via asexual budding, and they readily produce haploid gametes via meiosis, but these gametes are inviable. Gamete viability is measured experimentally by placing individual haploid spores produced by a certain diploid strain onto an agar plate and counting the proportion of the total that produce colonies after two days of incubation. Both *Saccharomyces* species normally produce 90% to 100% viable gametes, but hybrids between them produce only approximately 1% [9]. There are several potential causes of this gamete inviability: chromosomal rearrangements, sequence divergence interfering with meiosis, dominant genetic incompatibilities, and recessive genetic incompatibilities.

In many species, hybrid sterility is caused by chromosomal translocations that interfere with meiosis or prevent gametes from receiving a complete haploid set of genes [10]. Some of the sensu stricto *Saccharomyces* species differ from one another by as many as four translocations, and these can contribute to hybrid spore inviability [11]. However, other *Saccharomyces* species, such as the S. cerevisiae and S. paradoxus strains used in this paper, do not have any translocations [12]. Their genomes are colinear apart from four very small inversions, two on Chromosome VII and two on Chromosome X [13]. These inversions could potentially cause the gametes of F1 hybrids to be inviable if a crossover occurred within an inverted region, producing acentric and dicentric chromosomes, gene imbalances, or loss of function if a crossover occurred between inverted copies of an essential gene. However, crossovers within microinversions should be extremely rare and thus are unlikely to account for many gamete deaths.

Another type of chromosomal incompatibility, which is caused by simple sequence divergence rather than chromosome rearrangements, has been shown to be important in yeast hybrids. Reduced homology—sequence divergence—reduces rates of homologous recombination [14]. In yeast, chromosomes must recombine with their homologs, forming chiasmata, to ensure proper meiotic segregation [15]. Thus, the reason that S. cerevisiae and S. paradoxus F1 hybrids produce inviable gametes might simply be that their chromosomes cannot recombine during meiosis because their sequences are too diverged and so they do not segregate effectively. The rare viable gametes that are produced from a hybrid meiosis are highly aneuploid, as expected if chromosomes segregate randomly, and few of their chromosomes are recombinant [16,17]. Hunter et al. [16] succeeded in increasing F1 gamete viability 10-fold by artificially increasing the rate of recombination using mutants in the mismatch repair system.

In principle, genetic incompatibilities could cause gamete inviability in yeast F1 hybrids either by affecting some aspect of gamete production, such as meiosis or sporulation, or by allowing normal gamete production but then killing the hybrid gametes once formed.

To disrupt F1 hybrid gamete production, genetic incompatibilities must be dominant, because any incompatible loci would be heterozygous in an F1 yeast hybrid. In an earlier experiment [18], we found that making diploid F1 hybrids into allotetraploids completely restored the viability of their gametes, showing that sterilizing incompatibilities were absent. We therefore concluded that dominant genetic incompatibilities are not the cause of F1 gamete inviability.

Recessive genetic incompatibilities are heterozygous in diploid hybrids and so cannot directly affect gamete production itself, but they could kill hybrid haploid gametes after they have formed. Yeast gametes are produced in the form of spores that germinate into metabolically active gametes. If unfertilized, viable gametes divide via mitosis and form colonies. In contrast to higher organisms such as *Drosophila,* yeast gametes express their haploid genomes, so recessive genetic incompatibilities, hemizygous in haploids, could render inviable the gametes of F1 hybrids.

This paper describes a screen to detect these types of recessive speciation genes. Individual chromosomes were first transferred from S. paradoxus gametes into S. cerevisiae gametes, producing strains that were haploid except for a single disomic (and hybrid) pair of chromosomes. I then selected each for the loss of the native chromosome to test the ability of the foreign chromosome to substitute for it and maintain gamete viability (see Materials and Methods). In this way, I used individual chromosomes as small samples of genome and tested them for the presence of gamete-killing speciation genes.

## Results

Nine different S. paradoxus chromosomes were transferred into S. cerevisiae gametes, where they successfully replaced their S. cerevisiae homeologs. Therefore, none of these nine chromosomes contain speciation genes that are capable, individually, of killing hybrid gametes.

### Chromosome Transfers

**Figure 1:**
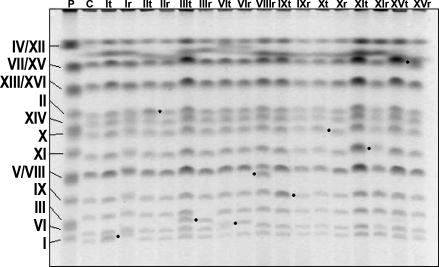
CHEF Gel Showing S. paradoxus Chromosome Transfers and Replacements in S. cerevisiae Gametes Negative digital image of a CHEF gel. Replaced chromosomes are indicated by an asterisk to the left of the S. paradoxus band. S. paradoxus Chromosomes I, III, VI. and VIII are a different size than their S. cerevisiae homeologs and can be readily identified. The remaining transfers were verified by quantitative image analysis of the high-intensity band produced by the disomic chromosome (see Materials and Methods). Lanes: P, S. paradoxus YDG749; C, S. cerevisiae YDG 780; It, S. cerevisiae haploid with additional (disomic) Chromosome I transferred from *S. paradoxus;* Ir, S. cerevisiae haploid with Chromosome I replaced by Chromosome I from *S. paradoxus;* etc.

I transferred Chromosomes I, II, III, VI, VIII, IX, X, XI, and XV from S. paradoxus gametes into S. cerevisiae gametes (see Materials and Methods and Figure 1). Candidate strains with the correct combination of genetic markers were initially karyotyped by PCR with species-specific primers for all possible chromosome ends from both species (64 in total; see Materials and Methods). I found that for two of the targeted chromosomes (I and IX), the first candidate strains tested were correct, whereas for other targeted chromosomes, many strains had to be screened before one with the correct karyotype was identified. The number of strains that had to be screened before the correct karyotype was found is shown in parentheses after the target chromosome number: I (one), II (18), III (eight), VI (seven), VIII (two), IX (one), X (nine), XI (four), and XV (five). The cause for rejection of all the failed candidate strains was that the PCR markers showed that other S. paradoxus chromosomes were transferred along with the targeted S. paradoxus chromosome. A linear regression found no significant relationship between the physical size of the target chromosome and the number of candidate strains that needed to be screened (regression on log-transformed values, *r* ^2^ = 0.14, 8 *df*, *p* = 0.32). Three chromosomes were targeted, but to date no correct transfers have been identified. These chromosomes (with the number of candidates screened in parentheses) are IV (six), XIII (eight), and XIV (11). The other four chromosomes were not been targeted either because they contain genes or markers used for the transfer method (V, *ura3, can1;* VII *cyh2*) or because no suitable auxotrophic marker has been identified in which to insert *URA3* (XII, XVI).

### Chromosome Replacements

S. paradoxus Chromosome VIII spontaneously replaced its S. cerevisiae homeolog before selection was applied with the drug 5-fluororotic acid (FOA). The other eight S. paradoxus chromosomes coexisted as disomes with their homeologs until the strains were replica-plated to FOA agar, which selected for the loss of the *URA3*-marked native chromosome (see Materials and Methods). All then produced FOA-resistant colonies that contained 15 S. cerevisiae chromosomes and one S. paradoxus chromosome, as confirmed by using species-specific PCR markers for the chromosome ends (see Materials and Methods). The rate of FOA-resistant colony formation varied greatly, presumably inversely reflecting the ability of the *URA3-*marked S. cerevisiae chromosome to segregate mitotically as a disome with its S. paradoxus homeolog. In order to quantify the rate, I counted the number of FOA-resistant colonies formed in a 10% sample of each plate's area. The number of colonies formed is given in parentheses after the disomic chromosome number: I (309), II (eight), III (27), VI (176), IX (128), X (282), XI (one), and XV (347). Again, the size of the disomic chromosome was not an accurate predictor of the rate of FOA-resistant colony formation (linear regression on log-transformed values: *r* ^2^ = 0.047, 7 *df, p* = 0.61).

### Confirmation of Transfer and Replacement of Targeted Chromosomes by Pulsed Field Gel Electrophoresis

**Figure 2:**
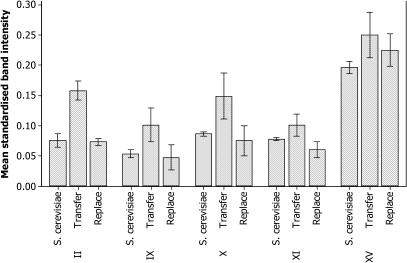
Results of Pulsed Field Gel Image Analysis The graph shows the mean standardized band intensities (a measure of the fraction of total cell DNA) for the five targeted S. paradoxus chromosomes that comigrate to the same bands as their S. cerevisiae homeologs. The error bars represent 95% confidence intervals. Four independent replicates were produced for Chromosomes II, IX, X, and XI. Ten independent replicates were produced for Chromosome XV. Raw images of all the gels as well as a table of all the image data are included in Figures S1–S5 and Table S1.

As described above, many candidate strains were rejected after PCR analysis of their karyotypes because they contained additional chromosomes to those targeted. Therefore, another, independent, karyotyping method was desirable as a quality control to confirm the PCR results. Pulsed field gel electrophoresis is a robust method that allows separation of the chromosomes on a gel and direct visualization of a strain's karyotype. Figure 1 shows a typical gel containing all the chromosome transfer and replacement strains. S. paradoxus Chromosomes I, III, VI, and VIII migrate in the gel at different rates to their S. cerevisiae homeologs and can be readily identified by the different bands they form. Thus, confirmation of the successful transfer and replacement of these targeted S. paradoxus chromosomes is easily verified (see Figure 1). S. paradoxus Chromosomes II, IX, X, XI, and XV migrate to the same band in the gel as their S. cerevisiae homeologs, so a different method is required to identify them on the gel. Transfer strains that are disomic for both species' Chromosome II, IX, X, or XI will have bands that contain twice the DNA and hence are twice as intense as those in normal *S. cerevisiae.* Chromosome XV migrates to the same band as VII, so a strain disomic for both species' XV chromosomes will have an XV/VII band that is 50% more intense than in normal *S. cerevisiae.* Many of these intensity differences can be detected by the naked eye ([16] and see Figure 1), but to obtain quantitative measurement of the DNA content of bands, at least four independent preparations of each strain were made, run out independently on separate gels, and analyzed using image analysis software (see Materials and Methods for details). The results of this analysis are summarized in Figure 2, and the raw images and image analysis data are presented in Figures S1–S5 and Table S1. The mean standardized intensities for normal S. cerevisiae chromosome bands II, IX, X, XI, and XV were compared to those in their respective transfer and replacement strains by one-way analysis of variance and post hoc Tukey test (see Materials and Methods). In each case, the band for the targeted transferred chromosome was significantly more intense than the same band in normal *S. cerevisiae,* showing that it was disomic and transfer had been successful (*p* < 0.001, *n* = 4 for Chromosomes II, IX, X, and XI; *p* < 0.05, *n* = 10, for Chromosome XV). The XV/VII band for the XV transfer strain was not found to be significantly more intense when only four preparations were analyzed because its intensity is expected to increase by only 50%, rather than by 100% for the others. Thus, six more preparations were made and analyzed to increase the statistical power of the test. The II, IX, X, and XI bands in the (FOA-resistant) chromosome replacement strains were significantly less intense than the band in each transfer strain, showing that the native chromosome had been lost and successfully replaced by the S. paradoxus chromosome. The XV chromosome replacement band was less intense than that in the XV chromosome transfer strain (Figure 2), but it was not statistically significant. However, given that the XV transfer strain formed FOA-resistant colonies at a higher rate than any other strain (see Chromosome Replacements, above) and that in all other transfer strains the first FOA-resistant colony tested was found to have lost the targeted native S. cerevisiae chromosome, it would be very unlikely that the XV replacement strain actually remained disomic. Rather, the lack of significant difference between the XV/VII doublets in the transfer and replacement strains probably reflects the lack of power of the test to detect the relatively small difference in intensity expected in the XV/VII band (see above).

### Test for Rogue Chromosome Transfer by Pulsed Field Gel Electrophoresis

I used the same pulsed field gels generated above to screen the transfer strains for additional (rogue) S. paradoxus chromosomes that might have been inadvertently transferred along with the targeted chromosome and also escaped the PCR screen. S. paradoxus I, VI, III, and VIII are readily identified by their size difference: none of these chromosomes were found (except in the strains in which each was targeted). All the other chromosomes except IV/XII were tested (described in Materials and Methods) to identify bands that were significantly more intense than in S. cerevisiae and that might therefore be disomic. For Chromosomes II, IX, X, XI, and XV, the brightest band identified was that of the targeted chromosomes, showing that the test successfully identifies known disomes (a positive control for the screen). For these chromosomes, the next brightest band was then identified for each strain, and the mean of four replicates was compared to the mean of the band in the four S. cerevisiae replicates by one-way analysis of variance. In no case was the brightest band significantly brighter than in normal S. cerevisiae (analysis not shown; all intensity data are presented as supplementary material). For Chromosomes V, XIV, XIII, XVI, and VII, in which no targeted transfers were successful, the mean of the brightest band was not significantly different from the mean of the band in S. cerevisiae. It is possible, but unlikely, that rogue disomes exist that escaped the PCR screen and also escaped the image analysis screen, either because their bands could not be quantified (IV and XII) or because they are in bands of two comigrating chromosomes and a third rogue does not increase the brightness sufficiently to be statistically significant with only four replicate samples (V, XIII, XVI, VII, and XV). However, the reliability of the PCR karyotype assay is ensured by the fact that the image intensity screen found no rogue chromosomes in the nine that can be identified with confidence.

## Discussion

In this paper, I have shown that nine of the 16 S. paradoxus chromosomes can substitute for their S. cerevisiae homeologs without causing death of the cells that contain them. Thus, none of the nine chromosomes contain speciation genes that are capable of killing vegetative hybrid gametes.

### Estimate of Total Number of Recessive Speciation Genes Causing F1 Gamete Inviability

Assuming that the chromosomes were sampled randomly with respect to the presence or absence of incompatible genes, and if loci that have incompatible alleles are randomly distributed across the genome, then we can calculate the maximum likely number of gamete-killing speciation genes on the unsampled seven chromosomes. If there was a single speciation gene, it would more likely than not avoid detection, because I sampled 43% of the genome, so it would have a probability of 0.57 (1 − 0.43) of falling on a chromosome that was not tested. But the probability of six genes that are capable of killing hybrid gametes being missed falls below the 5% boundary for statistical significance [*p* = (1 − 0.43)^6^ = 0.035], so we can be confident that there are between zero and five S. paradoxus genes that are incompatible with genes on the S. cerevisiae genome in such a way that they kill hybrid gametes that contain them*.*

### Contribution of Genes Causing Hybrid Gamete Inviability to F1 Hybrid Sterility

F1 hybrid sterility could have many possible causes, among them the gamete-killing recessive speciation genes screened for in this study. We can ask, therefore, if the sole cause of hybrid sterility was this type of genetic incompatibility, how many such genes would be required to reduce diploid F1 hybrid fertility to 1%? The simplest model for the evolution of genetic incompatibility [1,2] features two interacting genes, *A* and *B,* in two genetically identical geographically isolated populations. In one population, a new allele, *a,* is fixed, and in the other, a new allele, *b*. The evolved alleles *a* and *b* are incompatible with each other. A diploid F1 hybrid will have the genotype *AaBb* and will be viable and fertile if the incompatible alleles are recessive. Its gametes will have viable genotypes *AB, Ab,* and *aB* and the inviable genotype *ab.* So, a single recessive incompatibility can reduce fertility by no more than 25%, leaving 75% of gametes viable. To reduce fertility to 1%, the fertility of the yeast hybrid, a minimum of 16 incompatibilities are required [(1 − 0.25)^16^ = 1%]. This is a conservative estimate assuming the most powerful form of recessive speciation gene, a two-locus completely lethal incompatibility. If incompatibilities involve more than two loci, then they will affect fewer gametes (e.g., a three-locus incompatibility would only affect 12.5% of gametes), and if incompatibilities are not completely lethal, then more would be required to have a sterilizing effect. Five incompatibilities, the maximum that could reside on the unsampled chromosomes (see Estimate of Total Number of Recessive Speciation Genes Causing F1 Gamete Inviability, above) could only reduce F1 fertility to 24% (0.75^5^), more than 20 times higher than the actual F1 hybrid fertility of 1%. Thus, the sterility of yeast F1 hybrids cannot be explained solely by gamete-killing speciation genes.

An important assumption of this analysis is that the nine chromosomes tested represent a random sample of the genome. In addition to the nine chromosomes transferred, three chromosomes (IV, XIII, and XIV) were targeted but were not successfully transferred. This is not surprising: transferring single chromosomes by this method relies on rare random events—transfers of whole individual chromosomes between separate nuclei—that are very unlikely for large chromosomes like those unsuccessfully targeted [19]. Another possibility, though, is that these three chromosomes could not be transferred because they contain incompatible genes. Such an incompatible allele would need to be dominant (it would be heterozygous when transferred), but it would interact with recessives on the haploid genome. In organisms that have chromosomal sex determination, such as *Drosophila,* such dominant–recessive interactions are thought to cause Haldane's rule (that males are more affected than females by hybrid incompatibilities) [20]. X-linked genes are hemizygous in XY males, so recessive incompatibilities on the X chromosome interact with dominant incompatibilities elsewhere. Recessive–dominant interactions are a good explanation for Haldane's rule, but they are much less common than recessive–recessive incompatibilities [20,21]. So, we would not expect to find recessive–dominant incompatibilities without also finding recessive–recessive incompatibilities. Furthermore, recessive–dominant interactions cannot contribute to yeast speciation, because yeast lacks chromosomal sex determination and yeast cells are found only as either complete diploids or complete haploids. Thus, it is reasonable to assume that the nine chromosomes transferred are a random sample and that the three unsuccessful transfers failed by chance, rather than because they were prevented by recessive–dominant incompatibilities.

### Gamete-Killing Incompatibilities That Would Not Be Detected by the Assay

Incompatibilities between loci on homeologs of the same chromosome would not be detected because whole chromosomes (containing both compatible alleles) were transferred. However, yeast has 16 chromosomes, so the chance that a given speciation gene shares the same chromosome as the locus with which it interacts is only 1:16, on average. Moreover, incompatible loci on homeologs of same chromosome could kill the gametes produced by F1 hybrids only if they were far enough apart to be unlinked (so that the *ab* inviable genotype could be produced in gametes), further reducing their ability to contribute to hybrid sterility. Also able to escape detection are incompatibilities that cannot kill gametes by themselves but are lethal when combined with other such incompatibilities. However, these weak speciation genes can kill only a small proportion of gametes produced from an F1 hybrid (e.g., if two incompatibilities are required to be present in a gamete to kill it, only 6.25% of gametes will inherit both, leaving 93.75% of gametes viable). Similarly, complex interactions can kill fewer hybrid gametes than simple two-locus interactions (e.g., a three-locus incompatibility would affect only 12.5% of gametes).

### Other Recessive Speciation Genes That Would Not Be Detected by the Assay

Chromosomes were transferred between mature gametes, so incompatibilities that sterilize F1 hybrids by killing their gametes only before gamete maturation would escape detection. Such incompatibilities might exist in genes expressed specifically during spore germination or even in genes expressed specifically in the late stages of meiosis and spore production, because after meiosis I, many loci can be considered hemizygous and thus recessive incompatibility could be exposed. However, this group of genes is expected to be very small compared to all the genes required for vegetative growth, so there is no reason to expect to find many incompatibilities in germination if none are found in vegetative growth. Finally, genetic incompatibilities that reduce fitness but do not actually kill gametes can still contribute to speciation. F1 hybrid fertility would not be affected (because gametes would be viable, if unfit), but the low fitness of the resulting F2 hybrids could reduce their reproductive potential, contributing to reproductive isolation. Likewise, recessive incompatibilities that are only expressed in diploids, such as genes involved in meiosis, would not affect the fertility of F1 hybrids but, when homozygous in F2 hybrids, could render them sterile or inviable (hybrid breakdown).

### Conclusions

These results show that recessive Dobzhansky–Muller speciation genes are unlikely to be responsible for killing the gametes produced by S. paradoxus–S. cerevisiae F1 hybrids. While some types of recessive speciation genes cannot be detected by the assay used here, these are expected to be either rare or to kill only a small proportion of the gametes produced by F1 hybrids or only affect F2 hybrids. Thus, recessive speciation genes, if they exist at all, are surprisingly rare and are unlikely to be the major reason that the gametes produced by F1 hybrids are inviable.

So why are yeast F1 hybrids sterile? The most likely explanation is that of Hunter et al. [16], who showed that deleting the mismatch repair gene *MSH2* increased hybrid fertility 10-fold. They proposed that yeast hybrid sterility was caused by the failure of diverged homeologous chromosomes to recombine in meiosis, in part because of the action of the mismatch repair system. This failure leads to mis-segregation, lost chromosomes, and inviable aneuploid gametes [14,15,22]. The genomes of S. cerevisiae and S. paradoxus are about 15% diverged [23]. Recent work has shown that very low levels of divergence can also affect fertility [24,25]. Thus, it is likely that simple DNA sequence divergence is the major cause of sterility in F1 hybrids formed between S. cerevisiae and *S. paradoxus.*

## Materials and Methods

### Chromosome transfer and replacement.

The method was based on that of Chambers et al. [22] and Nilsson-Tillgren et al. [26] and uses a *kar1-Δ13* mutation [27] that prevents chromosome fusion after mating but allows individual chromosome transfer between nuclei [19]. S. cerevisiae strain YDG 780 *(alpha, ura3-d, cyh2r, can1r kar1-Δ13),* which is isogenic with S288c, was used to generate strains with different nutritional auxotrophies marking specific target chromosomes (see Protocol S1). These strains were allowed to mate overnight on YEPD agar with S. paradoxus haploid YDG 749 *(ho, MATa, ura3),* which is isogenic with N17 [28]. The mated cells were replica-plated to SC agar containing cycloheximide (3 mg/L) and canavanine (60 mg/L), to select against S. paradoxus haploids and hybrid diploids, and lacking the specific nutrient needed because of the auxotrophic marker on the target chromosome, to select for complementation of that marker by transfer of the wild-type S. paradoxus chromosome. Strains identified as disomic for the target chromosome (see Results and Protocol S1) were replica-plated to FOA-agar selecting for the loss of the *URA3-*marked S. cerevisiae target chromosome.

### Verification of chromosome transfer.

The complete chromosome complement of candidate strains was determined by PCR with species-specific primers for all possible chromosome ends from both species (64 in total; see Protocol S1 for primer sequences). Strains containing the correct chromosomes were then verified physically using pulsed field gel electrophoreses as described previously [16]. Transfer and replacement of Chromosomes I, III, VI, and VIII were confirmed visually; these chromosomes migrate differently on the gel according to which species they are from (see Figure 1 and Results). Transfer of the other five chromosomes was confirmed by quantitative measurement of the appropriate band intensity using the gel analysis plugin for the open source software ImageJ (Imaging Processing and Analysis in Java; http://rsb.info.nih.gov/ij). Bands of disomic chromosomes are much brighter than those of monosomic chromosomes. Four independent pulsed field gel preparations were made from each strain. Lanes were standardized by dividing the intensity of the target chromosome band by the sum of all the band intensities in the same lane except for the IV/XII band, which varies because of spontaneous changes in rDNA tandem repeat number. The mean of the four standardized intensities of the chromosome band of interest was then compared to the mean of the four standardized intensities of that band in *S. cerevisiae.* Six additional preparations were made of the XV transfer, replacement, and S. cerevisiae strains in order to increase the statistical power sufficiently to detect the relatively small difference in intensity between an XV/VII band containing three chromosomes (the transferred XV S. paradoxus chromosome and the normal XV/VII chromosomes) and an XV/VII band containing two chromosomes. Statistical comparisons were performed with Minitab 14 statistical software (http://www.minitab.com). The raw images and a table of image intensity data are provided in Figures S1–S5 and Table S1.

## Supporting Information

Figure S1 Raw tif Image File of Contour-Clamped Homogeneous Electrical Field (CHEF) Gel Referred to in Table S1
(300 KB TIF)

Figure S2 Raw tif Image File of CHEF Gel Referred to in Table S1
(300 KB TIF)

Figure S3 Raw tif Image File of CHEF Gel Referred to in Table S1
(6.9 MB KB TIF).

Figure S4 Raw tif Image File of CHEF Gel Referred to in Table S1
(6.9 MB KB TIF).

Figure S5 Raw tif Image File of CHEF Gel Referred to in Table S1
(6.9 MB KB TIF).

Protocol S1 Details of Strains and Methods (36 KB DOC)

Table S1 Microsoft Excel Spreadsheet of Band Intensity Data from the Images Supplied (85 KB XLS)

## Abbreviations

CHEF: contour-clamped homogeneous electrical field
FOA: 5-fluororotic acid
