# Supplementary material for: A Screen for Recessive Speciation Genes Expressed in the Gametes of F1 Hybrid Yeast
Source: PLoS Genet. 2007 Feb 16;3(2):e21. doi: 10.1371/journal.pgen.0030021 (PMC1797814; doi:10.1371/journal.pgen.0030021)
Supplement: Supplementary file 1 [file pgen.0030021.sd001.doc]

Supporting Information

Strains

*S. cerevisiae* strains are isogenic with S288c. Strains S1 and S96 (gifts from John McCusker, Duke University) were crossed with other strains as follows to insert the required alleles: *ura3Δ0* from strain BY4741 (Brachmann et al 1998) and *cyh2r* from strain M169 (a gift from Ed Louis, University of Nottingham). *kar1-*Δ13 was created using plasmid pMR1591, a gift from Mark Rose (Vallen et al, 1992). *can1r* alleles were derived by spontaneous mutation on synthetic medium containing canavanine, and the *URA3* disruption alleles were made by PCR-mediated transformation using plasmid pRS406 as a template (Brachmann et al 1998). A list of the strains used in this experiment follows:

*S. cerevisiae*

YDG 780 *ho MAT-*α *ura3Δ0* *cyh2r can1r kar1-Δ13*

*S. paradoxus*

YDG 749 *ho MAT-a ura3*

*Disomic strains*

YDG 941 (I) *ho MAT-*α *ura3Δ0* *cyh2r can1r kar1-Δ13 ade1::URA3/ADE1*

YDG 942 (II) *ho MAT-*α *ura3Δ0* *cyh2r can1r kar1-Δ13 tyr1::URA3/TYR1*

YDG 943 (III) *ho MAT-*α/*MAT-*a *ura3Δ0* *cyh2r can1r kar1-Δ13 thr4::URA3/THR4*

YDG 944 (VI) *ho MAT-*α *ura3Δ0* *cyh2r can1r kar1-Δ13 his2::URA3/HIS2*

YDG 945 (IX) *ho MAT-*α *ura3Δ0* *cyh2r can1r kar1-Δ13 his6::URA3/HIS6*

YDG 946 (X) *ho MAT-*α *ura3Δ0* *cyh2r can1r kar1-Δ13 met3::URA3/MET3*

YDG 947 (XI) *ho MAT-*α *ura3Δ0* *cyh2r can1r kar1-Δ13 met14::URA3/MET14*

YDG 948 (XV) *ho MAT-*α *ura3Δ0* *cyh2r can1r kar1-Δ13 ade2::URA3/ADE2*

*Chromosome substitution strains*

YDG 949 (I) *ho MAT-*α *ura3Δ0* *cyh2r can1r kar1-Δ13*

YDG 950 (II) *ho MAT-*α *ura3Δ0* *cyh2r can1r kar1-Δ13*

YDG 951 (III) *ho MAT-*a *ura3Δ0* *cyh2r can1r kar1-Δ13*

YDG 952 (VI) *ho MAT-*α *ura3Δ0* *cyh2r can1r kar1-Δ13*

YDG 953 (VIII) *ho MAT-*α *ura3Δ0* *cyh2r can1r kar1-Δ13*

YDG 954 (IX) *ho MAT-*α *ura3Δ0* *cyh2r can1r kar1-Δ13*

YDG 955 (X) *ho MAT-*α *ura3Δ0* *cyh2r can1r kar1-Δ13*

YDG 956 (XV) *ho MAT-*α *ura3Δ0* *cyh2r can1r kar1-Δ13*

YDG 965 (XI) *ho MAT-*α *ura3Δ0* *cyh2r can1r kar1-Δ13*

PCR

DNA for PCR templates was extracted using glass beads as described in (Burke et al, 2000). About 0.5ng template was used per 20ul reaction, with 250uM dNTPs, 10pmole primers and 0.3U of Taq polymerase. Reaction conditions were 40 cycles of 96ºC-55ºC-72ºC. The following primer pairs were used:

*S. paradoxus* I L: TTCCAGAGGACGGG, CATGTGTAGTTGGATCGTAG

*S. cerevisiae* I L: CCATTTAAAGGAGCCACA, GACTCTAATTGCATGCCA

*S. paradoxus* I R: CCTGTTCCGCCACTA, CACGACGTACTTGCACT

*S. cerevisiae* I R: TTAAAGAGACTACCCATCCC, CCGAATAGCCGCTCATA

*S. paradoxus* II L: GGTGCAGGTGTGCTA, AGGAGAAATAACCGATACC

*S. cerevisiae* II L: GCTGCTATTCGTTCTCTATC, CAAAATACCTGGTGCTTT

*S. paradoxus* II R: GGTTTGTCCAAGACCG, GGTGGCAATAACTACGGTA

*S. cerevisiae* II R: TAAGCACTTTTTCAACATTCAG, GTTGGGTCAGTCATCCTC

*S. paradoxus* III L: CAAGCCTTGGGATACAA, TAGTTCCAAGGCTTTCAG

*S. cerevisiae* III L: GAGGTGATTGGCAAAAAT, GAAACCTTTACACACTACAAAA

*S. paradoxus* III R: TTAGTCCTAAAGGCTACGC, ATTGACATGCCATTACTTTT

*S. cerevisiae* III R: TGGTTCTGAAAGCAACAT, CTGCAATCAAGGCTTAAA

*S. paradoxus* IV L: ACGTCGCTTAGGTCG, ATGGCCAACCCACT

*S. cerevisiae* IV L: CCACAAAGACCTTTTTAATG, AGGGTCGCAGAATCC

*S. paradoxus* IV R: AAGATCCGCGATAGATTT, AGACCAGGTATCTTCCATC

*S. cerevisiae* IV R: AGAACCTGGTAAGCTTGC, TAATGTTGCAGACTGATTTT

*S. paradoxus* V L: GAGACTCTCGAGCTCATG, AGCCCTCTTGTAAACAACT

*S. cerevisiae* V L: CGAACCTTCCGACAA, GCATCACCTAGTTCTTTCA

*S. paradoxus* V R: TCACAGACCTATTGGCA, TTACTCTTCGCTGGCA

*S. cerevisiae* V R: GAACAAACGGCCCTTAT, GAAGACGTTGGGAAGGT

*S. paradoxus* VI L: GGAGAAACTGTTCATGAAGA, ACATCTGTATGAGCCATAAAC

*S. cerevisiae* VI L: CCGCTTCTTGGGTAACT, TAGGTTCAGCACCGGT

*S. paradoxus* VI R: CCTCTTCTATCGAGACAATTT, TCTGCACTTGGGTGAAT

*S. cerevisiae* VI R: TATTGAGTATGAGAGCGTCC, ATCTTCATATGGAGCTTCG

*S. paradoxus* VII L: ACATAAGATAGTGGAATTGGAC, GAAGTATGGTGATGCACAG

*S. cerevisiae* VII L: AGGTGGATGACGTCG, CTTATCATCCGACGTGAA

*S. paradoxus* VII R: TAGAGCTAGGACGCGA, CTAGTTCGAGGAAATAGAGGA

*S. cerevisiae* VII R: TGTGAAGGGTGAAGTTTTAG, CTTATGCTCCTTTTTGTCC

*S. paradoxus* VIII L: CGCAACAAAAATCTGATT, AACGTTTTCATCATCAGC

*S. cerevisiae* VIII L: ATTGAGGAGACAGGGC, TCGAGGATTTTTGAGTTAGAT

*S. paradoxus* VIII R: TTAGTTTGGAGGACGGT, CAACGAGAGACGTGATAAT

*S. cerevisiae* VIII R: CTGCACATTAACAACAAGA, CTGTCTTTCCTCGGGA

*S. paradoxus* IX L: TGGAGAAACAAGACACG, TTATATAGCGTCTTCGACAAG

*S. cerevisiae* IX L: ATCGCTGTCACTTCACG, ACTTCATGCCACCTGTTG

*S. paradoxus* IX R: GAGGATGAAATAGGAGGTG, ATAGCAAATTGTCTGCG

*S. cerevisiae* IX R: GACAAAAAACAAGATAACAGAGAC, GATTTATCATGCTTTGGCT

*S. paradoxus* X L: AGTTTAATGCTGCTTTCG, CATCTCGAACCAACCTC

*S. cerevisiae* X L: GATCTTATTCAGTTGATCCTAAC, AAATTACTTGATAGACCGACC

*S. paradoxus* X R: CCCTTCCCTTGTCG, AACACCAAGACTTGCTAATAG

*S. cerevisiae* X R: AGCTTCACTACCCCTATCC, AGGTTGAGAT TGCCCTC

*S. paradoxus* XI L: CCTGAGTTTATTTTCGCA, TCACCCCTTGCCTGATAA

*S. cerevisiae* XI L: TGTGCTGGATTCCTTAGAC, GAAGGTGTTACCAAACAGC

*S. paradoxus* XI R: GAACTCGAAATAGCCAAGA, AAGATACTGTTCATTCTTAGAAGAC

*S. cerevisiae* XI R: GTTTGTGAAAAGGAAGCTC, CTGTTCATTTTTTGAACGTAG

*S. paradoxus* XII L: GCACGTCCAACCCCT, AGCGCCCGTATTAGCG

*S. cerevisiae* XII L: TCAAGTAATAATTTCATTGTGGA, GCCGGAAAACACCAGAT

*S. paradoxus* XII R: CAACAGTGATACCAAATCG, AATAATTGAATCCGTAACG

*S. cerevisiae* XII L: GCATGAATTATCTTCCAAA, GATGATTGAATCCGTGAC

*S. paradoxus* XIII L: ACAGAAGACAACATCCATTT, TGTCAGGATCAACGTTG

*S. cerevisiae* XIII L: CTCCACTTCATCAAGGAA, TCTTGTCGTAAACGGTCT

*S. paradoxus* XIII R: TTAGATTTTGCGAAGACC, TTCTTCTTATCCGACAAATC

*S. cerevisiae* XIII R: GACCAATCAGTGCTTCCT, GAAACAATGATTAGCACTTTG

*S. paradoxus* XIV L: GAGAACTCTACCAATGGG, AATCAGTCGTTAGTGAGCTAT

*S. cerevisiae* XIV L: GCTCGCATTTTAACAAGTAC, TTCAGTGTTCAGAGATTGC

*S. paradoxus* XIV R: GTTAGAACAAGGGCCAC, GTTGCCTTAGCCACG

*S. cerevisiae* XIV R: CATTGCAAGATTTCACAAG, CTGGCATATGACATTAGCTG

*S. paradoxus* XV L: CCGATTACACCAAGCGC, GCCACAGAGTTGAACTATAAAC

*S. cerevisiae* XV L: TGATTGAAGAAAGAGATTTGG, GTCATTATTCGAGCAAGGA

*S. paradoxus* XV R: AAGACAGCTTCCCACA, CGTGTCCGTTCTTGTC

*S. cerevisiae* XV R: GGATAAAGTATTATCAGAGTTT, TCCGAAGATATCTCCGAGT

*S. paradoxus* XVI L: GAGACCACCACCAGTATC, TGGCTCCAATTGGC

*S. cerevisiae* XVI L: CCAAGAGAACTTTTGTGAA, GAATTTCCAGACAATACGAT

*S. paradoxus* XVI R: AAAACCTTCCAGTTGATTTA, CTTCATCGTCTTCCTCA

*S. cerevisiae* XVI R: TCTATACGGTCAAGCTACT, GCATGGGCAAGGCAAC
